# Thoracic endovascular aortic repair (TEVAR) for an acute type A aortic dissection following transcatheter aortic valve replacement (TAVR)

**DOI:** 10.1016/j.jvscit.2024.101653

**Published:** 2024-10-24

**Authors:** Vy C. Dang, Paul Haddad, Ross G. McFall, Jennifer Klopfenstein, Michael J. Reardon, Maham Rahimi

**Affiliations:** a Texas A&M University College of Medicine, Bryan, TX; b Department of Cardiovascular Surgery, Houston Methodist Hospital, Houston, TX

**Keywords:** Thoracic endovascular aortic repair (TEVAR), Aortic stent graft, Aortic dissection, Type A aortic dissection, Transcatheter aortic valve replacement (TAVR)

## Abstract

An 86-year-old male with multiple medical comorbidities was referred for an elective transcatheter aortic valve replacement (TAVR) for severe symptomatic aortic stenosis with an ejection fraction of 35%. A self-expanding valve was deployed successfully, but intraoperative transesophageal echocardiography (TEE) confirmed an acute type A aortic dissection (ATAAD), necessitating immediate intervention. Multiple intraoperative imaging modalities were utilized for deployment of a conformable GORE TAG (cTAG) stent graft for thoracic endovascular aortic repair (TEVAR). This case demonstrates TEVAR as an effective, minimally invasive option for immediate repair of ATAAD as a complication of TAVR in a high-risk surgical patient.

An 86-year-old male with chronic congestive heart failure status-post implantable cardioversion-defibrillator placement, paroxysmal atrial fibrillation on apixaban, chronic kidney disease, obstructive sleep apnea, and diabetes mellitus was referred for an elective transcatheter aortic valve replacement (TAVR) for severe symptomatic aortic stenosis. Echocardiography demonstrated an ejection fraction of 35%. The patient’s past surgical history included an appendectomy, cholecystectomy, prostatectomy, and bilateral total knee replacements. He was considered high-risk for isolated surgical aortic valve replacement due to his age, frailty, and other risk factors. Results from the Society of Thoracic Surgeons risk score^1^ estimated an operative mortality rate of 6.47% and a morbidity and mortality rate of 13.4%. A 34-mm self-expanding valve (Medtronic Evolut PRO+, Medtronic) was placed via a transfemoral approach without pre- or post-dilation. There were no signs of a perivalvular leak on final arteriogram. However, a post-deployment intraoperative transesophageal echocardiogram (TEE) confirmed an iatrogenic acute TType A aortic dissection (ATAAD), requiring immediate intervention. Because no computed tomography angiography (CTA) was obtained after the intraoperative diagnosis of a dissection, the exact location of the entry tear in relation to the Medtronic Evolut PRO+ was unknown. In consideration of the patient’s options, operative risk, and clinical situation, a shared decision was made between the cardiac and vascular surgery teams to proceed with endovascular stenting of the ascending aorta. The patient provided written informed consent to the report of his case details and imaging studies.

Preoperative computed tomography (CT) scan of the aorta (Fig 1) allowed initial sizing of a 40 mm × 10 cm conformable GORE TAG (cTAG) stent graft. An outer curve length of >10 cm suggested that the 10-cm stent graft could be adequately deployed in the aortic arch. Intravascular ultrasound (IVUS) via right common femoral artery access confirmed the position of the wire in the true lumen and, in conjunction with intraoperative fluoroscopy and angiography, confirmed the origin of the innominate artery and the location of the coronary arteries in relation to the self-expanding valve. IVUS and angiography confirmed an acute type A_9_ aortic dissection. The stent graft body was delivered through the left common femoral artery access through which the transcatheter heart valve had been delivered. Right brachial artery access was obtained, and wire was passed through the innominate artery into the descending thoracic aorta. If the stent graft was inaccurately deployed and covered the innominate artery, bailout stenting was planned to preserve cerebral perfusion. Transcranial Doppler was also utilized to monitor cerebral perfusion intraoperatively. A marking catheter and IVUS confirmed the sizing of a 40 mm × 10 cm cTAG, in a 1:1 ratio without oversizing the stent graft. The patient was heparinized with a goal activated clotting time of ≥250. Rapid ventricular pacing and fusion was utilized to aid with accuracy of successful stent graft deployment in the ascending aorta (Fig 2). After deployment, a completion angiogram confirmed the lack of innominate artery coverage, endoleak, or evidence of visceral malperfusion. IVUS was also utilized to assess the visceral aorta, which did not show severe dynamic movement of the dissection flap across the celiac and superior mesenteric arteries.

**Fig 1:**
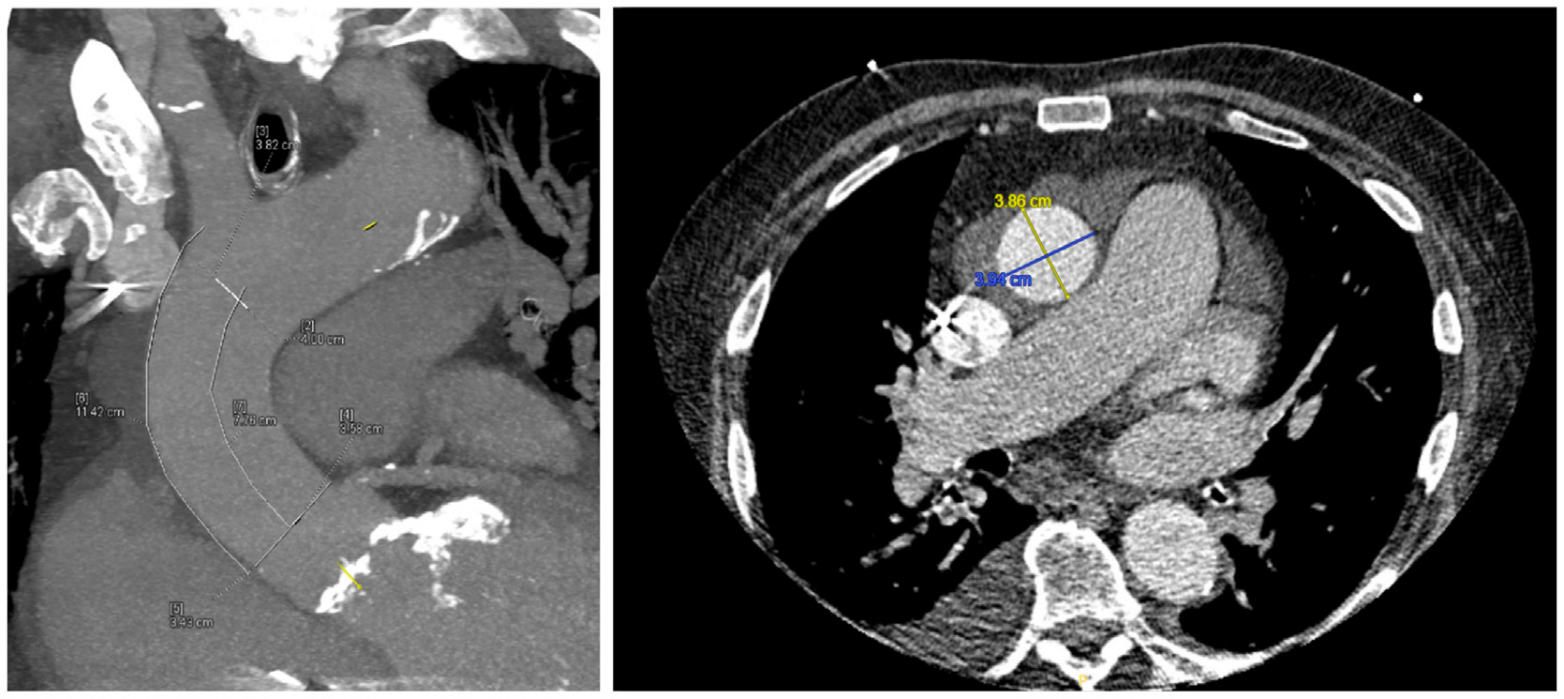
Preoperative computed tomography (CT) scan with labeled measurements of the ascending aorta.

**Fig 2:**
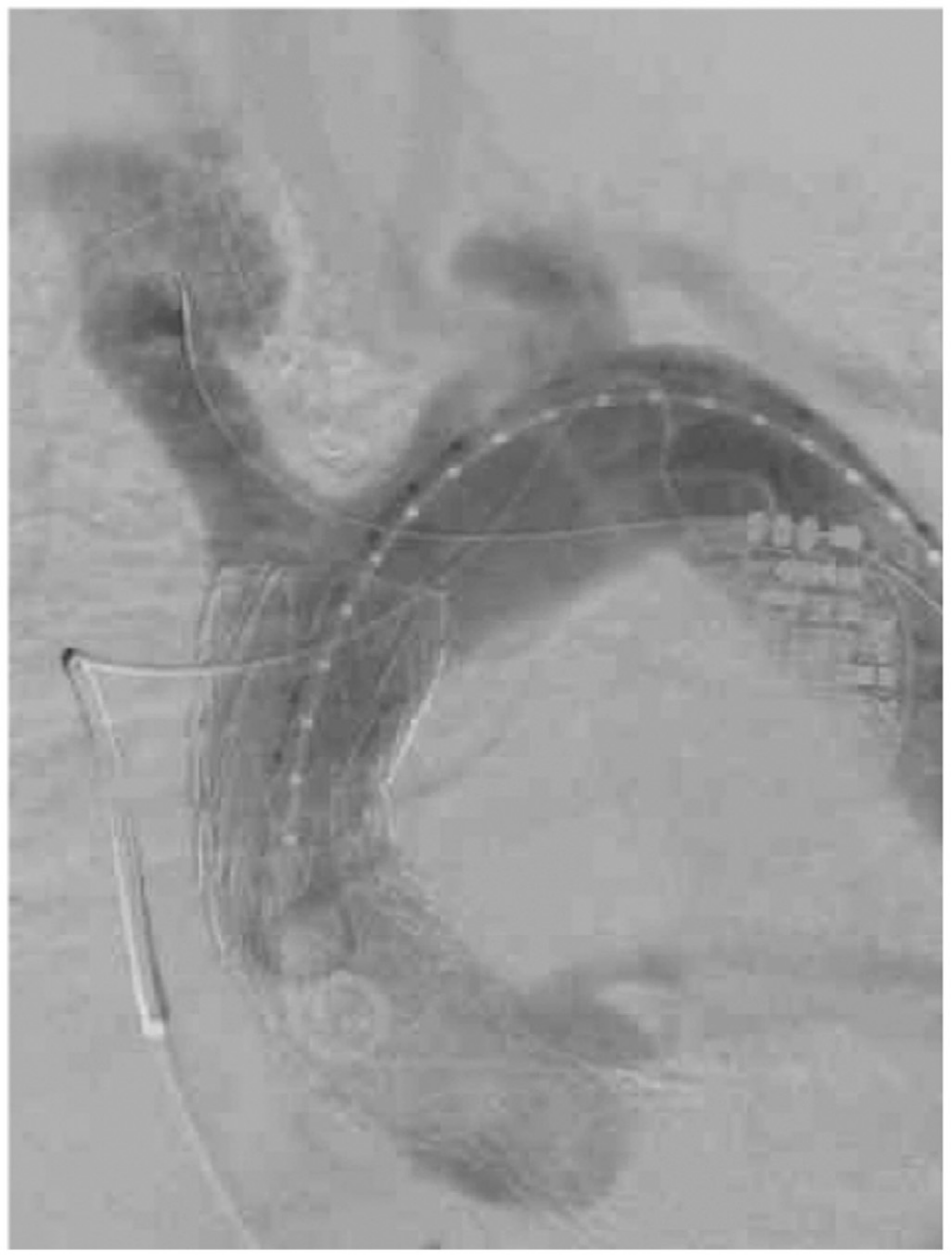
Deployment of the GORE cTAG stent graft visualized under intraoperative fluoroscopy.

The patient was transferred to the intensive care unit in a stable condition and remained in the intensive care unit for 3 days. Magnetic resonance angiography (MRA) on postoperative day 7 showed minimal false-lumen filling (Supplementary Video 1, online only), and the patient was discharged to an inpatient rehabilitation facility on postoperative day 10 without complications. The patient was followed in clinic after 2 months and was planned to have repeat imaging per our dissection protocol; however, unfortunately, he died months later from COVID-19 complications.

Iatrogenic aortic dissection is a rare complication of transfemoral TAVR, described in both intraoperative, early postoperative, and delayed postoperative periods, with a reported incidence of 0.1% to 1.9%.^2^, ^3^, ^4^, ^5^ Surgical management of TAVR-associated type A aortic dissections (TAAD), in particular, has included ascending aorta replacement, hemiarch vs total arch replacement, transcatheter heart valve explantation and replacement, and aortic root replacement, depending on the extent of the dissection. As TAVR patients are already considered high-risk for open surgical repair, conservative medical management has been the standard alternative in the event of an iatrogenic TAAD.^5^^,^^6^ Over the past 2 decades, there have been numerous publications describing the off-label use of endovascular stent grafts for TAAD in patients deemed unfit or high-risk for open repair.^7^ There have been fewer case reports describing the endovascular repair of TAVR-associated TAAD.^8^, ^9^, ^10^ Most recently, Eudailey and colleagues reported a successful repair of a subacute TAAD after transfemoral implantation of a 34-mm Evolut PRO (Medtronic) using a Valiant Navion stent (Medtronic) via suprasternal approach, which they describe to provide a more reliable bias to the lesser curve of the ascending aorta.^9^ We demonstrate the successful deployment of a TEVAR device in the ascending aorta and into the self-expanding valve via a transfemoral approach.

The GORE cTAG endoprosthesis is United States Food and Drug Administration-approved for thoracic endovascular aortic repair (TEVAR) of thoracic aneurysms and acute type B dissections. Appropriate anatomy is required, which entails adequate iliac/femoral access, aortic neck diameter of 16 to 42 mm, and ≥20 mm proximal landing zone without aneurysm or dissection.^11^ Indications for TEVAR have been largely limited to pathologies of the descending thoracic aorta. However, we describe its use here for treatment of ATAAD. The gold standard of ATAAD repair is open aortic replacement with a Dacron graft or frozen elephant trunk. Endovascular repair of ATAAD has been largely limited, as current anatomical challenges include a lack of a commercially available stent graft accessible in emergency settings that accommodates for the short length (<10 cm) from the sinotubular junction to the innominate artery, aortic diameter that may vary by phase in the cardiac cycle, and angulation of the ascending aorta.^12^ Obtaining an ideal landing zone is challenging, especially if the entry tear is anywhere but the middle portion of the ascending aorta.^13^ The stent graft must be deployed proximal to the entry tear but distal to the highest coronary artery; however, most entry tears of ascending aortic dissections occur within 2 cm of the sinotubular junction, near the ostia of the coronary arteries. Tears extending into the coronary arteries cannot be treated with TEVAR alone.^7^^,^^12^

In this case, the uncovered portion of the prosthetic valve served as the proximal landing zone for the cTAG stent-graft, allowing coverage of the entry tear without compromise of blood flow to the coronary arteries (Fig 3). Forward pressure was applied to the device after two-thirds of the stent graft was deployed to avoid covering the innominate artery. The difficulty in measuring the inner and outer curvature of the ascending aorta limits our ability to accurately measure the length by which the stent graft shortened; however, the completion angiogram demonstrated an unobstructed origin of the innominate artery.

**Fig 3:**
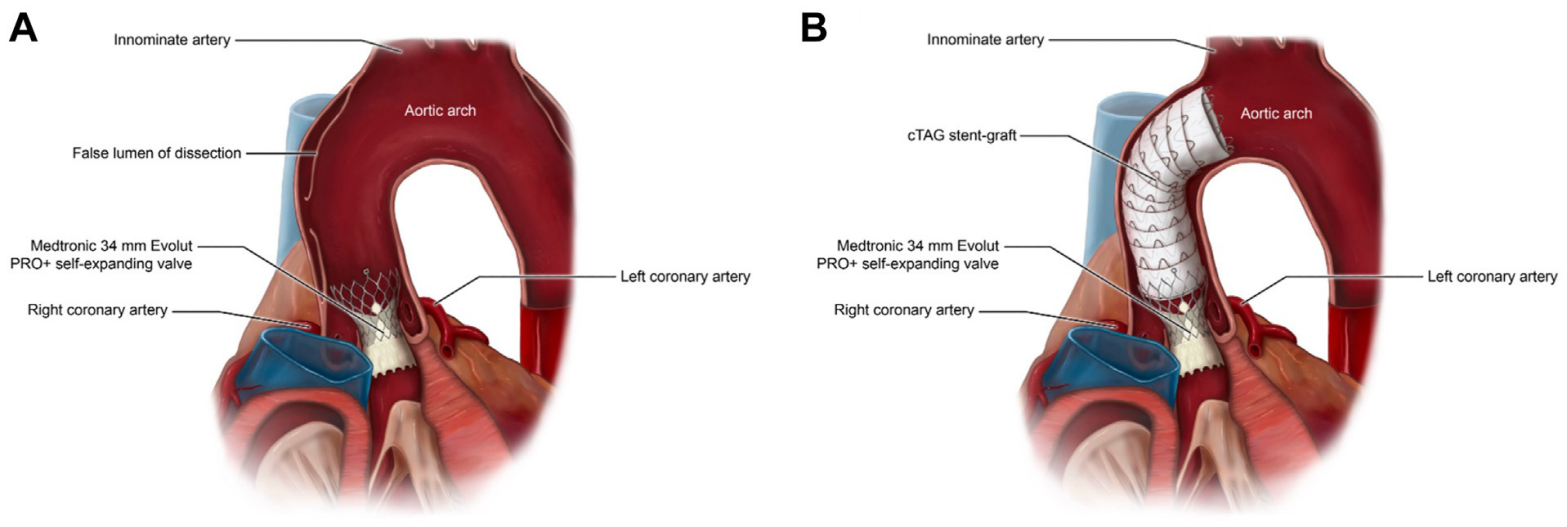
**(A)** Medtronic self-expanding valve and a type A_9_ dissection. Note: Although this figure demonstrates an entry tear in the mid-ascending aorta, the exact location of the entry tear was unknown. **(B)** GORE cTAG device deployed in the crown of the Medtronic self-expanding valve without covering the ostia of the coronary arteries.

We demonstrate the use of several imaging modalities for the surgical planning and execution of this case such as preoperative CT for sizing and intraoperative TEE, IVUS, angiography, and transcranial Doppler. Postoperative MRA was performed in this case due to the patient’s poor renal function; however, patients with normal renal function can undergo a postoperative dynamic CTA.

This case demonstrates TEVAR as an effective, minimally invasive option for immediate repair of ATAAD as a complication of TAVR in a high-risk patient.

## Disclosures

None.

## Appendix (online only)

Supplementary Video 1 (online only) Postoperative dynamic magnetic resonance angiography (MRA) demonstrates minimal false lumen filling.
